# Roles of Al_2_O_3_@ZrO_2_ Particles in Modulating Crystalline Morphology and Electrical Properties of P(VDF-HFP) Nanocomposites

**DOI:** 10.3390/molecules27134289

**Published:** 2022-07-04

**Authors:** Wenyue Zheng, Lulu Ren, Xuetong Zhao, Can Wang, Lijun Yang, Ruijin Liao

**Affiliations:** State Key Laboratory of Power Transmission Equipment & System Security and New Technology, School of Electrical Engineering, Chongqing University, Shapingba District, Chongqing 400044, China; zwybb0823@126.com (W.Z.); ren_lr969@163.com (L.R.); youthw27@163.com (C.W.); yljcqu@cqu.edu.cn (L.Y.); rjliao@cqu.edu.cn (R.L.)

**Keywords:** nanocomposites, crystalline morphology, interfacial relaxation, trap density, energy storage property

## Abstract

Polymer materials with excellent physicochemical and electrical properties are desirable for energy storage applications in advanced electronics and power systems. Here, Al_2_O_3_@ZrO_2_ nanoparticles (A@Z) with a core-shell structure are synthesized and introduced to a P(VDF-HFP) matrix to fabricate P(VDF-HFP)/A@Z nanocomposite films. Experimental and simulation results confirm that A@Z nanoparticles increase the crystallinity and crystallization temperature owing to the effect of the refined crystal size. The incorporation of A@Z nanoparticles leads to conformational changes of molecular chains of P(VDF-HFP), which influences the dielectric relaxation and trap parameters of the nanocomposites. The calculated total trapped charges increase from 13.63 μC of the neat P(VDF-HFP) to 47.55 μC of P(VDF-HFP)/5 vol%-A@Z nanocomposite, indicating a substantial improvement in trap density. The modulated crystalline characteristic and interfaces between nanoparticles and polymer matrix are effective in inhibiting charge motion and impeding the electric conduction channels, which contributes to an improved electrical property and energy density of the nanocomposites. Specifically, a ~200% and ~31% enhancement in discharged energy density and breakdown strength are achieved in the P(VDF-HFP)/5 vol%-A@Z nanocomposite.

## 1. Introduction

Sustainable development based on clean energy has attracted extensive attention worldwide, which concerns storing energy in environmentally friendly ways and increasing the efficiency of electrical energy conversion [1]. Numerous devices, including electrochemical batteries, supercapacitors and dielectric capacitors, have been developed for electrical energy storage technologies [2,3]. The dielectric film capacitors serve as the key unit in electric vehicles, high-voltage direct current (HVDC) transmission systems and pulse power systems owing to their fast charging-discharging rates, superior power density, long cycle life and great reliability [1,4]. Among the various dielectric capacitor materials, such as ceramics, mica and synthetic polymers, polymer-based dielectrics feature high breakdown strength, light weight, flexibility and facile preparation.

Currently, the energy density of benchmark commercial biaxially oriented polypropylene (BOPP) polymer film capacitors is only ~2 J cm^−3^, which is far behind the demand of modern power electronic systems [5]. The hexafluoropropylene (HFP) monomers employed to copolymerize with vinylidene fluoride (VDF) break apart the large crystals and decouple the ferroelectric domains, which promotes polarization and reduces the remnant polarization [6,7]. The resultant copolymers P(VDF-HFP) ferroelectric polymers are considered one of the most promising polymer dielectrics for high-energy-density film capacitors owing to their large dielectric permittivity (≈10 at 1 kHz) [5,8]. Nowadays, a variety of innovative inorganic nanofillers, such as ceramic particles with high dielectric permittivity, wide-bandgap nitrides and oxides, have been introduced into composite systems to fabricate capacitor films [5,8,9,10,11]. Recently, the core-shell-structured nanofillers formed by surface-functionalization have been well demonstrated as a promising route to mitigate the large contrast in dielectric permittivity between fillers and polymer phases [12,13]. Note that encapsulating zirconium dioxide (ZrO_2_, dielectric permittivity ≈25 and bandgap ≈5.8 eV) with aluminium oxide (Al_2_O_3_, dielectric permittivity ≈10 and bandgap ≈8.8 eV) takes advantage of the wide bandgap feature of Al_2_O_3_ shell to establish an insulating barrier and limit electrical conduction, which contributes to the improved energy density of nanocomposites [5,12,13].

The crystallization characteristic of polymer nanocomposites has been widely studied for decades. Kaur et al. proposed that the crystallite grains could prevent oriented crystals from disorienting, which increases the remnant polarization of nanocomposites [14]. Jiang et al. noted that the addition of nanoparticles induces smaller crystalline size of nanocomposites, which can suppress the leakage current at high electric field and enhance the discharge efficiency of the nanocomposites [15]. Rekik and Tsonos et al. reported that the nanoparticles play the role of nuclei, which can alter the kinetics of crystallization and influence the growth of crystalline phase in the nanocomposites [16,17]. Moreover, it is noteworthy that the molecules’ morphology and segmental dynamics influence the dielectric response and electric properties of nanocomposites [18].

Dielectric spectra and thermally stimulated depolarization currents (TSDC) analysis are both capable methods that can provide a wealth of information about molecule/ion/electron in dielectrics. Generally, two dielectric relaxation processes were observed in PVDF-based nanocomposites. First, the dielectric relaxation at high-frequency region is generally perceived as the intrinsic *α* orientational relaxation, which is associated with the segmental motion of the polymer matrix [19,20,21]. The second relaxation is considered to be originated from interfacial relaxation, also known as the Maxwell–Wagner-Sillars (MWS) polarization. Zhou et al. [19] proposed that the interfacial relaxation is related to the charge diffusion over neighboring filler interfaces. Stavrakas et al. [20] attributed the mechanism to dipolar relaxation in the crystalline phase or in the intermediate region between crystalline and amorphous phases. Wu et al. [22] ascribed the MWS polarization to the accumulated charge carriers at the interfaces between the nanofillers and polymer matrices. However, the interfacial relaxation mechanism in PVDF-based composites still remains unsettled.

In this work, Al_2_O_3_@ZrO_2_ (A@Z) core-shell nanoparticles were produced. The presence of A@Z nanoparticles influences the crystalline morphology and mechanical flexibility of nanocomposites. Dielectric loss (*ε″*) and conductivity of the P(VDF-HFP)/nanoparticles nanocomposites were tested over a wide temperature range. Furthermore, the TSDC measurement were employed to quantify the interfacial polarization effect on trap parameters of the nanocomposites. The resulting P(VDF-HFP) nanocomposite embedded with 5 vol% A@Z nanoparticles exhibits synergetic improvement in physicochemical, dielectric and energy storage performance. The correlations between the relaxation processes and discharged energy density of nanocomposites are discussed based on the interfacial microstructures.

## 2. Results and Discussion

### 2.1. Microstructure Characterization of the Nanocomposites

**Figure 1:**
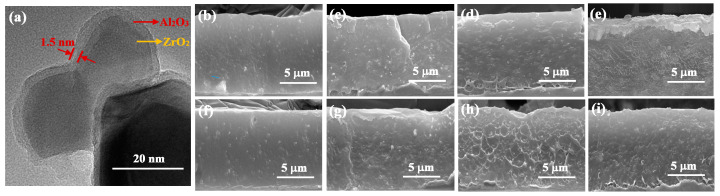
(**a**) TEM image of the A@Z nanoparticles, and SEM photographs of the P(VDF-HFP) nanocomposites loaded with (**b**) 1 vol% (**c**) 3 vol% (**d**) 5 vol% (**e**) 7 vol% of ZrO_2_ nanoparticles and (**f**) 1 vol% (**g**) 3 vol% (**h**) 5 vol% (**i**) 7 vol% of A@Z nanoparticles, respectively.

As illustrated in transmission electron microscope (TEM) image in Figure 1a, a clear core-shell structure of A@Z with a homogenous and compact shell layer is observed. The thin Al_2_O_3_ shell with thickness of ~1.5 nm is coated on the surface of ZrO_2_ core with a diameter of ~40 nm. Cross sectional scanning electron microscopy (SEM) photographs of the P(VDF-HFP)/Zr and P(VDF-HFP)/A@Z nanocomposites with different filler volume fractions are presented in Figure 1b–i. The thickness of the nanocomposite films is well controlled to be ~10 μm. Owing to the polar surface of metallic oxides [12,22,23], the ZrO_2_ and A@Z nanoparticles are well dispersed in the polymer matrix, showing no apparent filler aggregation on a large scale.

### 2.2. Thermal Characterization of the Nanocomposites

**Figure 2:**
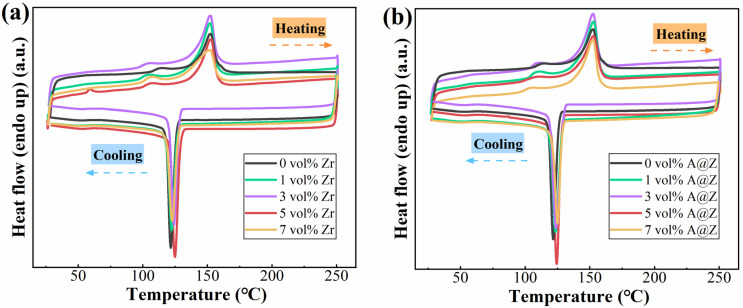
DSC curves of (**a**) the P(VDF-HFP)/Zr and (**b**) the P(VDF-HFP)/A@Z nanocomposites with a varied volume fraction of fillers.

Differential scanning calorimetry (DSC) curves of the neat P(VDF-HFP), P(VDF-HFP)/Zr and P(VDF-HFP)/A@Z nanocomposites are shown in Figure 2. A melting peak can be detected in the heating scan of all nanocomposites. The crystallinity (*X*_c_) of nanocomposites can be calculated via the following expression:

(1) $$X_{c}=\frac{\Delta H_{m}}{\left(1-\phi \right)\Delta H_{0}}\times 100\%$$

where Δ*H*_m_ is the enthalpy of fusion derived from melting peaks, *ϕ* is the mass ratio of the nanoparticle, Δ*H*_0_ is the heat of fusion for 100% crystalline P(VDF-HFP), which is reported to be 104.7 J/g [24]. As summarized in Table 1, the P(VDF-HFP)/5 vol%-A@Z nanocomposites present the largest *X*_c_ of 23.2%, compared with the values of 15.0% and 22.5% for the neat P(VDF-HFP) and P(VDF-HFP)/5 vol%-Zr nanocomposites, respectively. These results can be understood based on the intensified interaction among different molecules and increased heterogeneous nucleation points caused by the A@Z nanoparticles [9,24].

According to the cooling scan of DSC measurements, the crystallization temperature (*T*_c_) of the composites is increased with addition of the nanoparticles. As shown in Table 1, the *T*_c_ are 121.7 °C, 124.0 °C, and 124.3 °C for the neat P(VDF-HFP), P(VDF-HFP)/5 vol%-Zr, and P(VDF-HFP)/5 vol%-A@Z nanocomposites, respectively. It is believed that the nanoparticles act as nucleating agents and physical barriers to block the motion of polymer chains, making the nanocomposites crystallize at higher temperatures [15].

### 2.3. Mechanical Properties of the Nanocomposites

**Figure 3:**
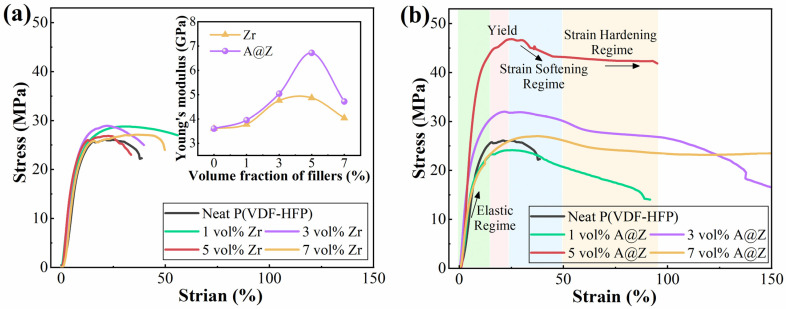
Stress–strain curves of (**a**) the P(VDF-HFP)/Zr and (**b**) the P(VDF-HFP)/A@Z nanocomposites with varied volume fraction of fillers. Young’s modulus of the P(VDF-HFP)/nanoparticle nanocomposites are shown in the inset of Figure 3a.

The stress–strain behavior and Young’s modulus results are illustrated in Figure 3a,b. As shown in Figure 3b, taking the P(VDF-HFP)/5 vol%-A@Z as an example, the stress-strain curve has four distinct regimes [25]: elastic regime, yield regime, softening regime and hardening regime. In the elastic regime, the stress increases nearly linearly with increasing applied strain. Upon reaching the yield point, the stress shows a slight decrease, suggesting that the material enters the softening region. Further deformation of the P(VDF-HFP)/nanoparticle nanocomposites causes an increase in strain hardening.

The relationship between the Young’s modulus and breakdown strength for the P(VDF-HFP)/nanoparticle nanocomposites can be described via Equation (2) [26,27],

(2) $$E_{b}≅0.6\sqrt{\frac{Y}{2\varepsilon_{0}\varepsilon_{r}}}$$

where *Y* is the Young’s modulus, *E*_b_ is the breakdown field. The Young’s modulus of nanocomposites determines the electromechanical failure caused by the coulombic force under an applied field. As shown in the inset of Figure 3a, the maximum Young’s modulus of the P(VDF-HFP)/A@Z nanocomposites is 6.72 GPa at the filler content of 5 vol%, which is 86.7% and 38.0% higher than that of the neat P(VDF-HFP) (3.60 GPa) and P(VDF-HFP)/5 vol%-Zr (4.87 GPa), respectively. It is thus reasoned that interfaces created by A@Z nanoparticles may improve the electromechanical behavior of the nanocomposites under a high electrical field [26]. However, the Young’s modulus and mechanical flexibility of nanocomposites were reduced when more nanoparticles were introduced, which may result in filler aggregation and induce the micro-crack sites at the interfaces between the fillers and polymer matrix [27].

### 2.4. Leakage Current Density of the Nanocomposites

**Figure 4:**
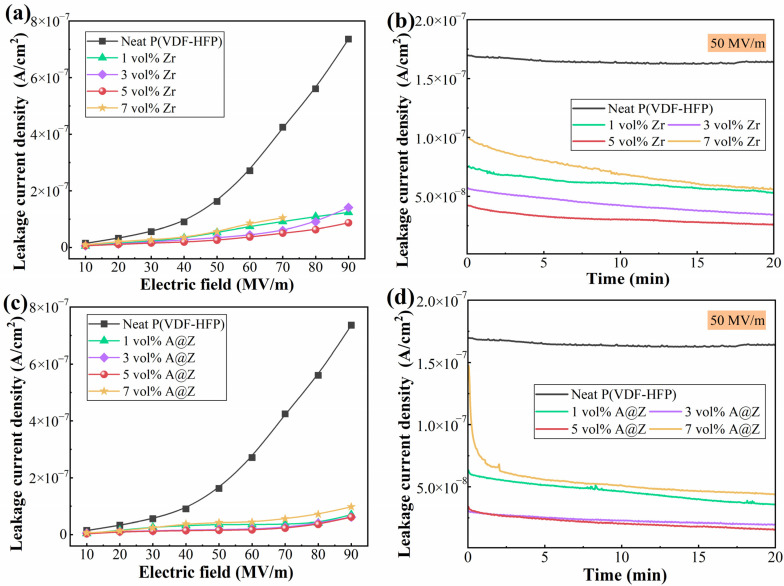
Leakage current density of the P(VDF-HFP)/Zr and P(VDF-HFP)/A@Z nanocomposites at varied electric field (**a**–**c**) and a typical electric field of 50 MV/m (**b**–**d**).

In general, leakage current is mainly caused by the movement of charge carriers such as ionic impurities or electrons injected from electrodes [21]. It has been demonstrated that the leakage current density plays a dominant role in determining the electrical conduction and energy loss of dielectrics. It is evident in Figure 4a that the leakage current density of the P(VDF-HFP)/Zr nanocomposites is minimized at a filler content of 5 vol%. For instance, the leakage current density measured at 50 MV/m decreases from 1.62 × 10^−7^ A/cm^2^ of the neat P(VDF-HFP) to 2.60 × 10^−8^ A/cm^2^ of P(VDF-HFP)/5 vol%-Zr (Figure 4b). The reduction in leakage current is mainly associated with the organic/inorganic interfaces, which may serve as trapping centers to hinder carrier transportation [9,28]. Taking advantage of the excellent insulation property of the Al_2_O_3_ layer, the P(VDF-HFP)/5 vol%-A@Z nanocomposite displays the minimum leakage current density of 1.54 × 10^−8^ A/cm^2^ at 50 MV/m, as shown in Figure 4c,d, which is 90.5% and 40.8% lower than neat P(VDF-HFP) and P(VDF-HFP)/5 vol%-Zr, respectively.

### 2.5. Dielectric Breakdown Strength of the Nanocomposites

**Figure 5:**
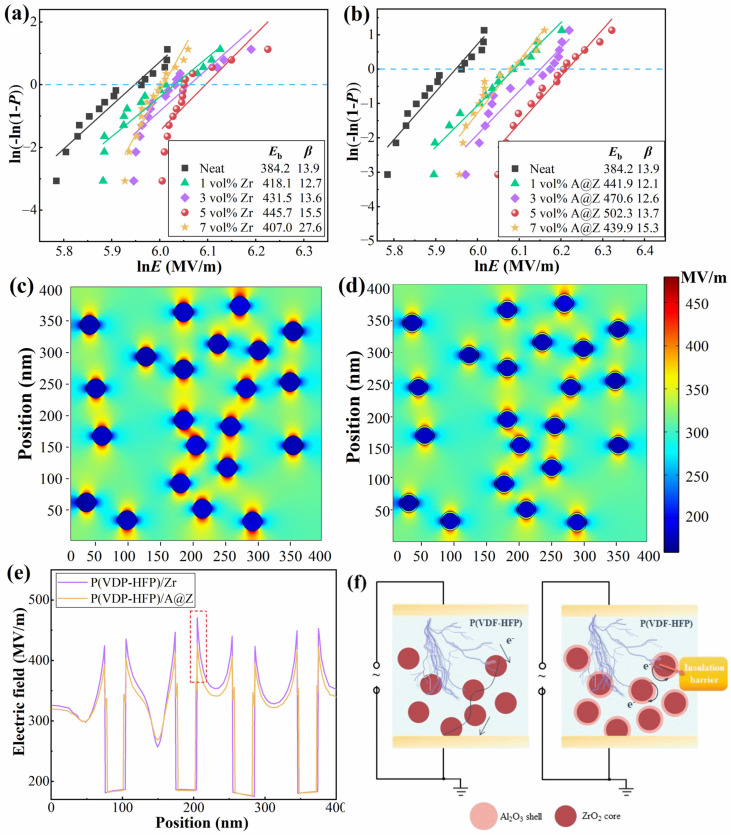
Breakdown strength of (**a**) the P(VDF-HFP)/Zr and (**b**) the P(VDF-HFP)/A@Z nanocomposites. Numerical simulations of local electric field distribution for (**c**) the P(VDF-HFP)/Zr and (**d**) the P(VDF-HFP)/A@Z nanocomposites. (**e**) Simulated local electric field near nanoparticles/matrix interfaces of the P(VDF-HFP)/Zr and P(VDF-HFP)/A@Z nanocomposites. (**f**) Schematic of the internal conduction channels for the P(VDF-HFP)/Zr and P(VDF-HFP)/A@Z nanocomposites.

The dielectric breakdown strength of nanocomposites is analyzed based on a two-parameter Weibull statistic model [9]:

(3) $$P(E)=1-\exp{\left(-(E/E_{b})\right)}^{\beta }$$

where *P* is the cumulative probability of dielectric failure, *E* is the measured discrete breakdown strength, *E*_b_ is the Weibull characteristic breakdown strength and *β* is the shape parameter which reflects the dispersion degree of the data. As depicted in Figure 5a,b, all the P(VDF-HFP)/nanoparticle nanocomposites exhibit high *β* values (>10), indicating a high electrical stability of the samples. The *E*_b_ of the P(VDF-HFP) nanocomposites is increased with the doping amount of ZrO_2_ and A@Z nanoparticles from 0 vol% to 5 vol%, and then decreased when the doping nanoparticles further reach 7 vol%. At the optimized content of 5 vol%, the *E*_b_ of P(VDF-HFP)/A@Z nanocomposite is 502.3 MV/m, which is much higher than that of P(VDF-HFP)/Zr nanocomposite (445.7 MV/m) and neat P(VDF-HFP) (384.2 MV/m). The breakdown fields of the neat P(VDF-HFP), P(VDF-HFP)/Zr and P(VDF-HFP)/A@Z nanocomposites exhibit the same trend as that of the Young’s modulus, as presented in the inset of Figure 3a. It agrees well with the Equation (2) that the high Young’s modulus favors the improvement of the breakdown strength of nanocomposites.

The finite element simulations are carried out under 300 MV/m to further clarify the influence of Al_2_O_3_ shell on the electric field distribution in nanocomposites. As shown in Figure 5c, an obvious electric field distortion occurs around the nanoparticles due to the large difference in dielectric permittivity and conductivity between ZrO_2_ and P(VDF-HFP) matrix [29,30]. Thus, the adjacent nanoparticles may form conducting channels and generate a potential partial breakdown in the nanocomposite [31], as illustrated in Figure 5f. Remarkably, the local electric field near the A@Z nanoparticles in P(VDF-HFP)/A@Z nanocomposite (Figure 5d) is lower and less distorted compared with the P(VDF-HFP)/Zr nanocomposite. In Figure 5e, the results show that the maximum local electric field of P(VDF-HFP)/A@Z nanocomposites is 427.2 MV/m, which is about 9.2% lower than that in P(VDF-HFP)/Zr nanocomposites (i.e., 470.6 MV/m), suggesting that the Al_2_O_3_ shell layer successfully impedes the propagation of conducting channels and modulates the local inhomogeneous electrical field. As a result, the P(VDF-HFP)/A@Z nanocomposites show a larger breakdown field at the same filling content.

### 2.6. Energy Storage Properties of the Nanocomposites

**Figure 6:**
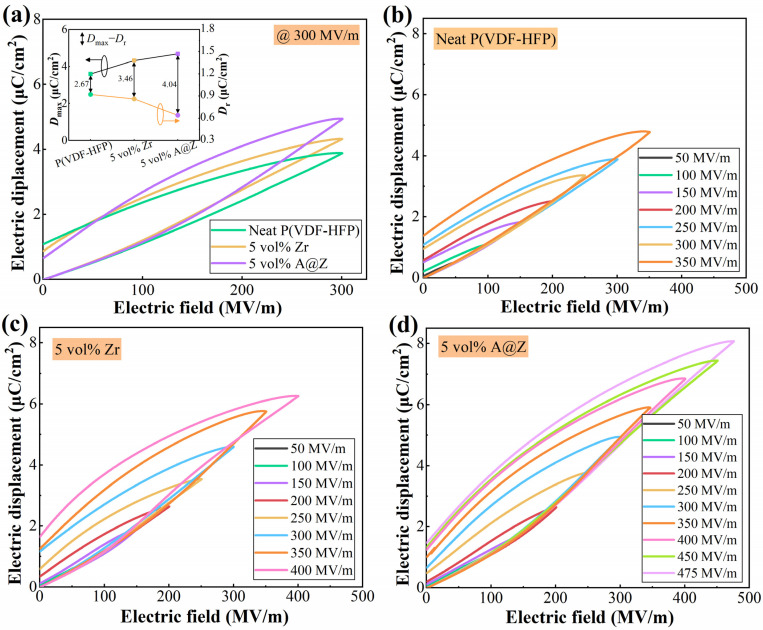
(**a**) *D*-*E* loops at 300 MV/m of the neat P(VDF-HFP), P(VDF-HFP)/5 vol%-Zr and P(VDF-HFP)/5 vol%-A@Z nanocomposites. *D*-*E* loops of (**b**) the neat P(VDF-HFP), (**c**) nanocomposites with 5 vol% of ZrO_2_ and (**d**) 5 vol% of A@Z nanoparticles. The variation of maximum displacement (*D*_max_), remnant displacement (*D*_r_) and *D*_max_ − *D*_r_ values are shown in the inset of Figure 6a.

The charge–discharge efficiency (*η*) is an important metric of polymer materials, as the heat generated by unreleased energy is detrimental to the performance and reliability of capacitors. The energy loss of P(VDF-HFP), including those from electrical conduction and polarization hysteresis caused by irreversible dipoles, can be manifested directly by the remnant displacement (*D*_r_), i.e., the electric displacement at zero electric field [26,28]. As shown in the insets of Figure 6a, the *D*_r_ at 300 MV/m decreases from 0.92 C/cm^2^ of the neat P(VDF-HFP) to 0.86 C/cm^2^ of P(VDF-HFP)/Zr nanocomposite with 5 vol% of ZrO_2_ and further decreases to 0.64 C/cm^2^ of P(VDF-HFP)/5 vol%-A@Z nanocomposite. The incorporation of nanoparticles suppresses the polymer recrystallization and brings about a decline in the crystallite size of nanocomposites [9,26]. The polymer crystals with smaller sizes facilitate the dipole orientation and inhibit the hysteresis loss [9,13,26]. Consequently, the incorporation of A@Z nanoparticles can significantly mitigate the reduction of *η* with applied field, e.g., the *η* of P(VDF-HFP)/5 vol%-A@Z nanocomposite is 71.4% at 300 MV/m compared to 51.4% of the neat P(VDF-HFP) and 64.2% of the P(VDF-HFP)/5 vol%-Zr nanocomposite.

**Figure 7:**
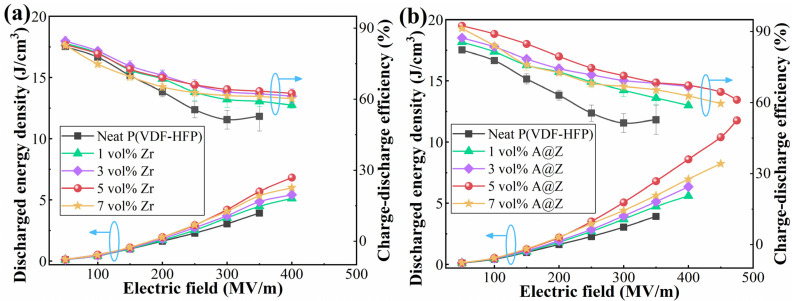
Discharged energy density and charge–discharge efficiency of (**a**) the P(VDF-HFP)/Zr and (**b**) the P(VDF-HFP)/A@Z nanocomposites with varied volume fraction of fillers.

The discharged energy density (*U*_d_) of the neat P(VDF-HFP), P(VDF-HFP)/Zr and P(VDF-HFP)/A@Z nanocomposites are calculated from the *D*-*E* loops (Figure 6b–d) and summarized in Figure 7a,b. As plotted in Figure 7b, the maximum *U*_d_ of the P(VDF-HFP)/A@Z nanocomposites can reach up to 11.8 J/cm^3^ at the filler content of 5 vol%, which is 202.6% and 49.4% higher than neat P(VDF-HFP) (3.9 J/cm^3^) and P(VDF-HFP)/5 vol%-Zr nanocomposite (7.9 J/cm^3^), respectively. This could contribute to the synergetic increase in *E*_b_ and *η* as well as the decrease in leakage current density [8].

### 2.7. Dielectric Loss of the Nanocomposites

**Figure 8:**
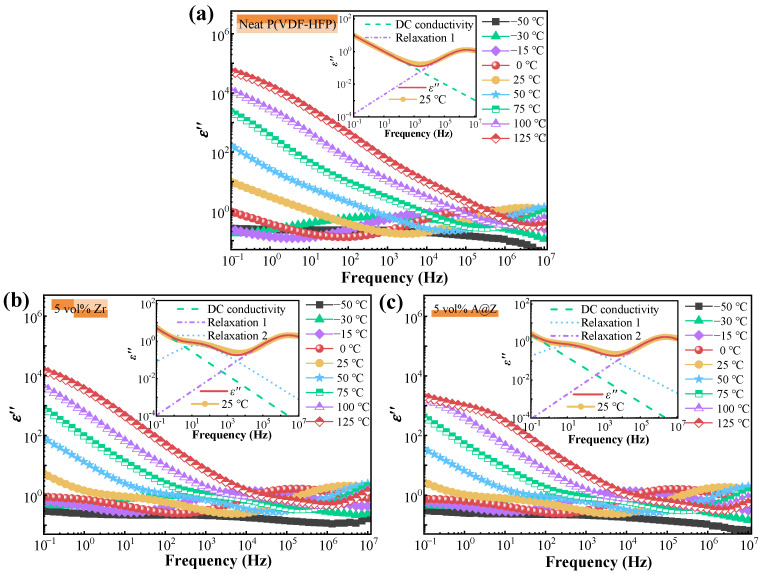
The spectra of *ε*″ versus frequency for (**a**) the neat P(VDF-HFP), (**b**) the P(VDF-HFP)/5 vol%-Zr and (**c**) the P(VDF-HFP)/5 vol%-A@Z nanocomposites at different temperatures. The *ε*″ − *f* at 25 °C fitted by H–N equation are shown in the inset of (**a**–**c**).

The dielectric loss (*ε*″) versus frequency (*ε*″ − *f*) of the neat P(VDF-HFP), P(VDF-HFP)/5 vol%-Zr and P(VDF-HFP)/5 vol%-A@Z nanocomposites at various temperatures are shown in Figure 8. It is evident that *ε*″ increases with increasing temperatures due to the accelerated segmental motions at high temperatures [32,33]. In the low-frequency interval, *ε*″ of all nanocomposites decreases linearly with the increasing frequency, which is a typical DC conduction process [32,34]. DC conduction is a result of localized charges jumping to neighboring sites and forming continuous connected networks, allowing the conduction current throughout the entire physical dimensions of the samples [33,35].

In Figure 8a–c, relaxation 1 over the high-frequency range is associated with the reorientation of the dipoles in the nanocomposites [33]. Interestingly, as illustrated in Figure 8b and c, a new relaxation process, i.e., relaxation 2 is introduced in both the P(VDF-HFP)/Zr and P(VDF-HFP)/A@Z nanocomposites, while it is absent in the neat P(VDF-HFP) (Figure 8a). As shown in the insets of Figure 8, the *ε″* − *f* at 25 °C is fitted by Havriliak–Negami (H–N) equation to reveal this new character [36]:

(4) $$\varepsilon^{\prime \prime }=\left(\varepsilon_{s}-\varepsilon_{\infty }\right){\left(1+2{\left(\frac{f}{f_{0}}\right)}^{\alpha }\cos\left(\frac{\pi \alpha }{2}\right)+{\left(\frac{f}{f_{0}}\right)}^{2\alpha }\right)}^{-\beta /2}\sin\left(\beta \varphi \right)$$

(5) $$\varphi =\arctan\left(\frac{{\left(\frac{f}{f_{0}}\right)}^{\alpha }\sin\left(\frac{\pi \alpha }{2}\right)}{1+{\left(\frac{f}{f_{0}}\right)}^{\alpha }\cos\left(\frac{\pi \alpha }{2}\right)}\right)$$

where *ε*_s_ and *ε*_∞_ are the unrelaxed and relaxed values of the relaxation for dielectric permittivity, respectively, Δ*ε* = *ε*_s_ − *ε*_∞_ is the dielectric strength of the relaxation, *α* and *β* (0 < *α*, *β* < 1) are the shape parameters which describe the symmetric and asymmetric broadening of the relaxation peak, respectively, and *f*/*f*_0_ takes the place of the product of relaxation time *τ* and angular frequency *ω*. Since the *ε*″ − *f* of the nanocomposites contains the DC conduction term as well as multiple relaxations, Equation (4) can be modified as:

(6) $$\varepsilon^{\prime \prime }=k_{0}+\sum_{k=1}^{n}\left[\left(\varepsilon_{ks}-\varepsilon_{k\infty }\right){\left(1+2{\left(\frac{f}{f_{k0}}\right)}^{k\alpha }\cos\left(\frac{\pi \alpha }{2}\right)+{\left(\frac{f}{f_{k0}}\right)}^{2k\alpha }\right)}^{-k\beta /2}\sin\left(k\beta \varphi \right)\right]$$

where *k*_0_ is the component coefficient of DC conduction, *k* is the order of relaxation processes. The strong nanoparticles/matrix interfacial barrier effect gives rise to relaxation 2 and decreases the hopping distance of charge carriers. Thus, the charge carriers possess reciprocating movement and can barely induce percolation paths in the nanocomposites [32,33]. The fitted parameters for the samples are listed in Table 2. It can be seen that the P(VDF-HFP)/5 vol%-A@Z nanocomposite has longer relaxation times compared to the neat P(VDF-HFP) and P(VDF-HFP)/5 vol%-Zr nanocomposite, indicating that the incorporation of A@Z nanoparticles modulates the hopping mechanism and restrains the motion of the charge carriers.

**Figure 9:**
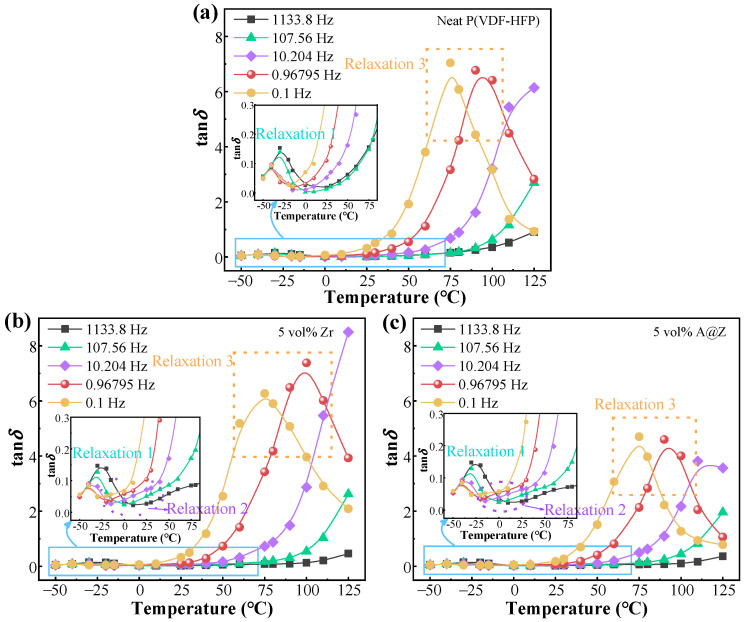
The spectra of tan*δ* versus temperature for (**a**) the neat P(VDF-HFP), (**b**) the P(VDF-HFP)/5 vol%-Zr and (**c**) the P(VDF-HFP)/5 vol%-A@Z nanocomposites at different temperatures.

In order to figure out the dynamic relaxation processes, the temperature dependences of the relaxations are presented in Figure 9. The peak 1 observed at the low-temperature range corresponds to the dipolar relaxation in the amorphous phase [20,21]. The relaxation 2 around 25 °C can be attributed to the nanoparticles/matrix interfaces, which is absent in neat P(VDF-HFP) of Figure 9a [16]. It is worth noting that the relaxation 3 covered by the DC conduction processes at low frequency in Figure 8 can be observed in the dielectric spectrum, varying with temperature, as shown in Figure 9.

### 2.8. AC Conductivity of the Nanocomposites

**Figure 10:**
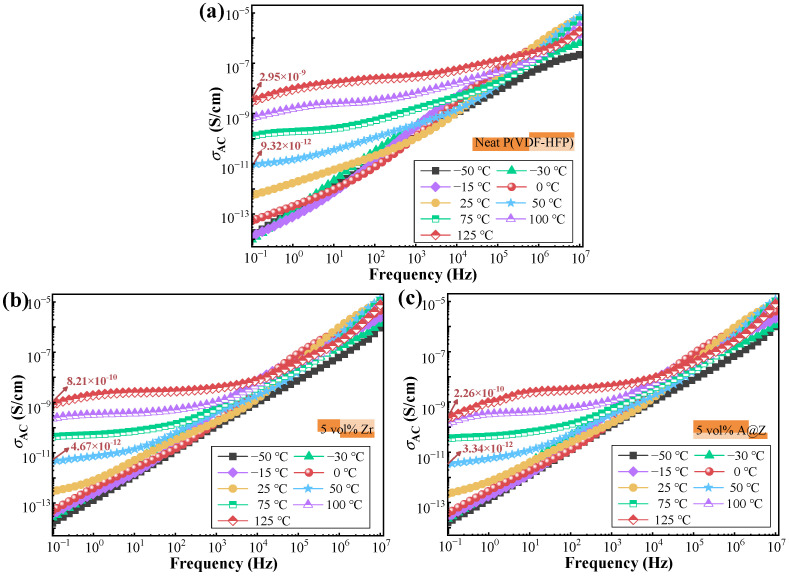
The spectra of *σ*_AC_ versus frequency for (**a**) the neat P(VDF-HFP), (**b**) the P(VDF-HFP)/5 vol%-Zr and (**c**) the P(VDF-HFP)/5 vol%-A@Z nanocomposites at different temperatures.

In order to further study the conduction mechanism, the AC conductivity (*σ*_AC_) of the P(VDF-HFP)/nanoparticle nanocomposites at various temperatures is given as a function of frequency in Figure 10. The plateau-like behavior observed at the low-frequency range of all the samples is defined as DC-like conductivity (*σ*_DC_) [29,31]. The regime at higher frequency is characterized by a frequency-dependent conductivity, which manifests that the *σ*_AC_ results from the MWS contributions and conforms to the dipolar relaxation modes [30]. The A@Z nanoparticles are effective in hindering electrical conduction, e.g., the *σ*_AC_ of P(VDF-HFP)/5 vol%-A@Z nanocomposite is 2.26 × 10^−10^ S/cm at 125 °C and 10^−^^1^ Hz, which is lower than the P(VDF-HFP)/5 vol%-Zr nanocomposite (8.21 × 10^−10^ S/cm) and the neat P(VDF-HFP) (2.95 × 10^−9^ S/cm), respectively. It has been reported that the wide bandgap feature of the Al_2_O_3_ shell may create higher energy barriers and limit the electrical conduction; therefore, the dielectric loss from DC-like conductivity and molecular relaxations is obviously lowered, as presented in Figure 8 [12,13,37].

### 2.9. TSDC Analysis of the Nanocomposites

**Figure 11:**
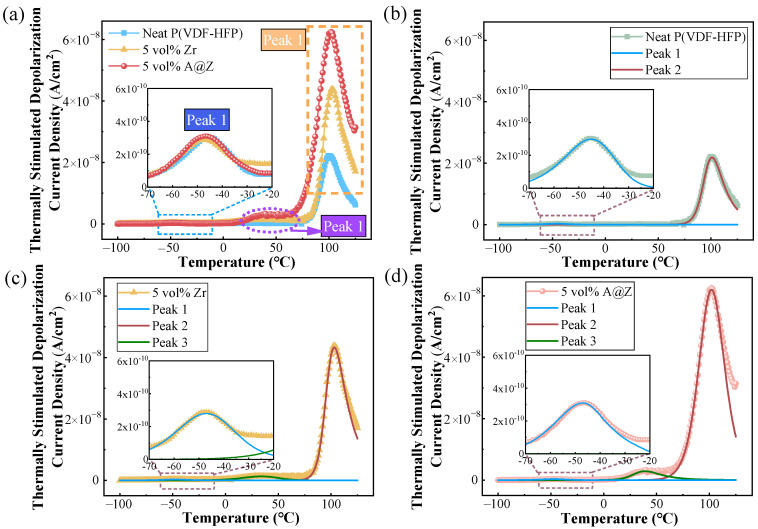
(**a**) TSDC curves of the neat P(VDF-HFP), P(VDF-HFP)/5 vol%-Zr and P(VDF-HFP)/5 vol%-A@Z nanocomposites. The TSDC fitting of (**b**) the neat P(VDF-HFP), (**c**) the P(VDF-HFP)/5 vol%-Zr and (**d**) the P(VDF-HFP)/5 vol%-A@Z nanocomposites.

The reduction in conductivity denotes a higher level of trap density arisen from the encapsulation of the Al_2_O_3_ shell, which can be further validated by the TSDC measurements [12,38]. As shown in Figure 11, multimodal Gaussian fitting is carried out to fit and split TSDC peaks of the nanocomposites for investigating various molecular relaxation mechanisms. The peak 1 observed at ~−45 °C is correlated with glass rubber transition and it can be attributed to the dipolar relaxation in the amorphous phase [20,21]. The TSDC peaks 2 at ~100 °C is originated from the interfacial relaxation due to charges accumulated in the amorphous/crystalline interfaces [20,39]. Obviously, the nanocomposite loaded with 5 vol% A@Z nanoparticles displays the most vigorous peak 2, confirming that the strong interfacial effect was caused by the addition of A@Z nanoparticles. Furthermore, it is inferred that the incorporated nanofillers can act as extensive carrier trap sites, as manifested by the occurrence of peak 3 at ~50 °C.

The trap site density of different TSDC peaks can be quantified based on the trapped charges, which are proportional to the intensity of TSDC curves [12,40]. As summarized in Table 3, the charges *Q*_1_, *Q*_2_ and *Q*_3_ corresponding to the TSDC peak 1, 2 and 3, respectively, were obtained, and we found that the total trapped charges *Q*_T_ (*Q*_T_ = *Q*_1_ + *Q*_2_ + *Q*_3_) increases from 13.63 μC of the neat P(VDF-HFP) to 29.20 nC of P(VDF-HFP)/5 vol%-Zr and 47.55 μC of P(VDF-HFP)/5 vol%-A@Z nanocomposites. It is believed that the presence of A@Z nanoparticles induces more trap sites at the interfaces of ZrO_2_/Al_2_O_3_ and Al_2_O_3_/P(VDF-HFP) in comparison to the uncoated ZrO_2_ nanoparticles, and thus the quantity of restricted charges is increased [13,19].

**Figure 12:**
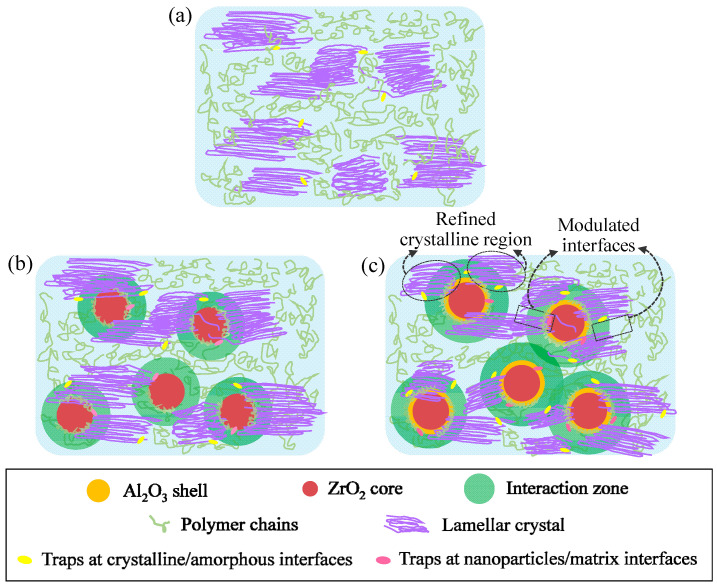
Schematic of the trap sites in (**a**) the neat P(VDF-HFP), (**b**) the P(VDF-HFP)/Zr and (**c**) the P(VDF-HFP)/A@Z nanocomposites.

The schematic of the interfacial microstructures and crystalline morphology in the neat P(VDF-HFP), P(VDF-HFP)/Zr and P(VDF-HFP)/A@Z nanocomposites are displayed in Figure 12a–c, respectively. From the morphological point of view, the interfaces between the amorphous and crystalline phase of P(VDF-HFP) or between the amorphous phase and the doped nanoparticles are of low density, leading to the formation of charge traps [41,42]. As depicted in Figure 12a, the trap sites at the crystalline/amorphous interfaces are attributed to the incorporation of nanoparticles that lead to conformational changes of polymer chain segments [43]. Haneef and Min reported that, under the steric hindrance of nanoparticles, the spatial overlap between the adjacent molecular chains determines the intermolecular coupling and thereby introduces more traps at the crystalline/amorphous interfaces of P(VDF-HFP)/nanoparticle nanocomposites [35,44].

The interfaces between the nanoparticles and the amorphous phase of P(VDF-HFP) (interaction zone) can also generate traps, as depicted in Figure 12b,c. The width of the interaction zone is shorter than the mean free path of charges, restraining the movement of the carriers [41,42]. In comparison with pristine ZrO_2_, the A@Z nanoparticles provide a larger interaction zone, which is beneficial to the increase in the trap density in the P(VDF-HFP)/A@Z nanocomposite [45]. Therefore, more free charges could be trapped in the interfaces between the nanoparticles and the amorphous region.

Concurrently, the crystallization characteristics of the nanocomposites as well as the interaction between nucleation agents and molecular chains may modulate the physicochemical and electrical properties of the nanocomposites [45,46]. According to the DSC measurement (Figure 2 and Table 1), the enhanced *T*_c_ of the P(VDF-HFP)/nanoparticle nanocomposites is the result of crystalline domain refinement effect [24]. It is known that the smaller size the crystalline domain is, the lower the free volume of the amorphous phase and the more crystalline/amorphous the interfaces are in the nanocomposites [24,26]. Since the mean free path of charge carriers in crystal region is much shorter than that in the amorphous region [47], the quantity of charges with restricted mobility in the P(VDF-HFP)/A@Z nanocomposite is increased, which may address the smaller leakage current density of the P(VDF-HFP)/A@Z nanocomposite than the neat P(VDF-HFP) and P(VDF-HFP)/Zr nanocomposites [9,24,48]. Low conduction current is beneficial to improved *E*_b_, *η* and *U*_d_ of the P(VDF-HFP)/A@Z nanocomposite. These results provide direct insights into the decreased energy loss and increased energy density of nanocomposites.

## 3. Materials and Methods

### 3.1. Materials and Sample Preparation

P(VDF-HFP) (Kynar Flex 2801) with 10% HFP was purchased from Arkema. ZrO_2_ (~40 nm) nanoparticles were purchased from US Research Nanomaterials, Inc. *N*, *N*-dimethylformamide (DMF), aluminum sulfate octadecahydrate, formic acid and ammonium formate were purchased from Sigma-Aldrich.

**Figure 13:**
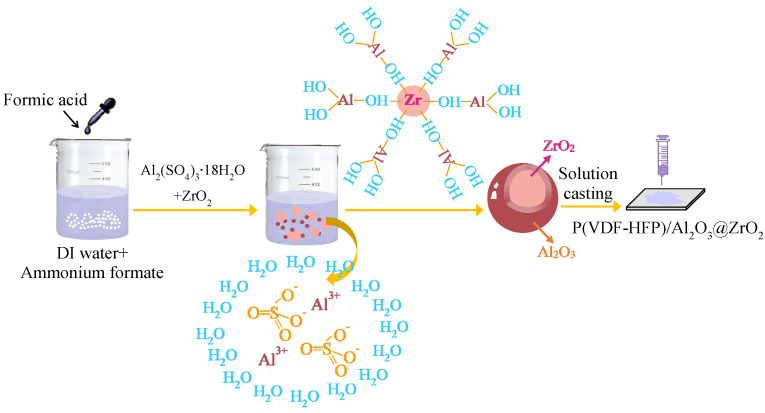
The fabrication process of the A@Z core-shell nanoparticles and the P(VDF-HFP) nanocomposites.

As presented in Figure 13, a nano-layer of Al_2_O_3_ was coated on ZrO_2_ nanoparticles via modified sol-gel method and the P(VDF-HFP) nanocomposites were fabricated via solution casting method. First, 0.1 mole of ammonium formate was dissolved into 500 mL deionized water under vigorous magnetic stirring. A certain amount of formic acid was added to adjust the mixture’s pH to 4.6. Then, 1 g ZrO_2_ powders and 1.10 g aluminum sulfate octadecahydrate (Al_2_(SO_4_)_3_⋅18H_2_O) were mixed with the resulting buffer solution. The above mixture was stirred at 70 °C for 5 h. Afterwards, the suspended particles were centrifuged and washed with deionized water at least 10 times. Finally, the solid sample was collected and heated at 600 °C for 10 h to obtain the core-shell structure A@Z nanoparticles (ZrO_2_ core, Al_2_O_3_ shell). The A@Z nanoparticles and P(VDF-HFP) powders were proportionally (0, 1, 3, 5 and 7 vol%) dispersed in DMF under ultrasonicating for 1.5 h. The suspension was cast on glass slides, followed by drying at 70 °C for 12 h in an air-circulating oven. After subsequently heating at 200 °C for 5 min, the samples were quenched in ice water. Finally, the samples were annealed at 105 °C for 24 h to remove solvent residue. The P(VDF-HFP) nanocomposites filled with raw ZrO_2_ nanoparticles, abbreviated as P(VDF-HFP)/Zr, were prepared in the same manner.

### 3.2. Sample Characterization

TEM images of A@Z nanoparticles were obtained by ThermoFisher Scientific instrument (Waltham, MA, USA). SEM images of the P(VDF-HFP) nanocomposites were characterized using Quattro S instrument. A Q100 DSC instrument (TA Instruments) was used to conduct DSC measurements. All samples were heated from 30 °C to 250 °C at a rate of 10 °C/min under nitrogen atmosphere. After holding at the state (250 °C) for 5 min to eliminate the thermal history, the samples were cooled down to 30 °C at the same rate. Electric displacement-electric field loop (*D-E* loop) tests were conducted on a PK-CPE2020-AI-20 kV high-voltage test system (PolyK Technologies, Philipsburg, PA, USA). Dielectric breakdown strength was measured with a DC voltage ramp of 500 V/s using a Trek 610E as the voltage source. The electric field distribution of P(VDF-HFP)/Zr and P(VDF-HFP)/A@Z nanocomposites were simulated by finite element model using COMSOL. An electric field of 300 MV/m was vertically applied from top to bottom of the model while the bottom is set to be the ground. Dielectric spectra were obtained using Novocontrol CONCEPT 80, with temperature ranging from −50 °C to 125 °C and frequency from 10^−1^ to 10^7^ Hz. TSDC measurements were carried out in a Delta oven and the current was measured by a 6517B electrometer. Detailed measuring steps are as follows:

(a) Placing the sample between electrodes and heating up to 180 °C.
(b) Applying the electric field of 200 V for 20 min to generate dielectric polarization.
(c) Cooling the sample to −100 °C rapidly under the applied field, where all the dipole/ionic motion is completely frozen.
(d) Connecting the sample to a short circuit condition for 10 min.
(e) Heating up to 125 °C at a linear rate and measuring the depolarization current as a function of temperature.

## 4. Conclusions

In conclusion, P(VDF-HFP) nanocomposites filled with ZrO_2_ and Al_2_O_3_@ZrO_2_ nanoparticles were developed. With the encapsulation of the wide-bandgap Al_2_O_3_ shell, the crystallinity and crystallization temperature are improved owing to the refined crystal size, which leads to suppressed leakage current density and AC conductivity. The presence of A@Z nanoparticles intensifies the intermolecular coupling and increases the area of crystalline/amorphous interfaces of the nanocomposites. The change in the molecules’ morphology and segmental motion in interfaces of the nanoparticles/amorphous phase modulate the charge hopping behavior, thus a new interfacial relaxation process is generated. The trapped charges during relaxation processes of P(VDF-HFP)/A@Z nanocomposite are increased, demonstrating that more trap sites are induced, which accounts for the stronger capability in inhibiting charges motion in the nanocomposites. Consequently, the P(VDF-HFP)/5 vol%-A@Z nanocomposite exhibits a substantial enhanced discharged energy density of 11.8 J/cm^3^. This study provides a new understanding for the improved dielectric and energy storage properties of the nanocomposites and also offers a promising strategy for the design and fabrication of the dielectrics for capacitors.

## Figures and Tables

**Table 1:** DSC results of the P(VDF-HFP)/Zr and P(VDF-HFP)/A@Z nanocomposites.

Volume Fraction of Fillers (vol%)	P(VDF-HFP)/Zr		P(VDF-HFP)/A@Z	
	*T*_c_ (°C)	*X*_c_ (%)	*T*_c_ (°C)	*X*_c_ (%)
0	121.7	15.0%	-	-
1	122.4	21.6%	123.5	21.9%
3	122.7	21.7%	123.8	22.0%
5	124.0	22.5%	124.3	23.2%
7	124.9	20.6%	125.0	21.2%

**Table 2:** The relevant parameters of H–N equation fitting for the neat P(VDF-HFP), P(VDF-HFP)/5 vol%-Zr and P(VDF-HFP)/5 vol%-A@Z nanocomposites.

Sample	*k*_0_	*α*_0_	*τ*_m1_	Δ*ε*_1_	*α*_1_	*β*_1_	*τ*_m2_	Δ*ε*_2_	*α*_2_	*β*_2_
Neat P(VDF-HFP)	3.12	0.50	5.06 × 10^−8^	5.11	0.59	1	*-*	*-*	*-*	*-*
P(VDF-HFP)/5 vol%-Zr	1.19	0.63	5.41 × 10^−8^	7.68	0.63	1	0.008	2.40	0.59	0.97
P(VDF-HFP)/5 vol%-A@Z	0.62	0.59	6.91 × 10^−8^	6.60	0.65	1	0.016	2.92	0.50	0.98

**Table 3:** The relevant parameters of TSDC fitting for the neat P(VDF-HFP), P(VDF-HFP)/5 vol%-Zr and P(VDF-HFP)/5 vol%-A@Z nanocomposites.

Sample	*Q*_1_ (μC)	*Q*_2_ (μC)	*Q*_3_ (μC)	*Q*_T_ (μC)
Neat P(VDF-HFP)	0.18	13.45	*/*	13.63
P(VDF-HFP)/5 vol%-Zr	0.17	27.73	1.30	29.20
P(VDF-HFP)/5 vol%-A@Z	0.18	44.91	2.46	47.55

## Data Availability

Not applicable.

## Sample Availability

Samples of the nanocomposites are available from the authors.
